# Zinc deficiency enhances sensitivity to influenza A associated bacterial pneumonia in mice

**DOI:** 10.14814/phy2.15902

**Published:** 2024-01-01

**Authors:** Radha Gopal, Egemen Tutuncuoglu, Veli Bakalov, Karla Wasserloos, HuiHua Li, David Lemley, Louis J. DeVito, Nicholas J. Constantinesco, Douglas S. Reed, Kevin J. McHugh, Baskaran Chinnappan, Alexis R. Andreas, Abigail Maloy, Daniel Bain, John F. Alcorn, Bruce R. Pitt, Ata Murat Kaynar

**Affiliations:** ^1^ Department of Pediatrics UPMC Children's Hospital of Pittsburgh Pittsburgh Pennsylvania USA; ^2^ The Clinical Research, Investigation, and Systems Modeling of Acute Illness (CRISMA) Center University of Pittsburgh Pittsburgh Pennsylvania USA; ^3^ Department of Critical Care Medicine University of Pittsburgh Pittsburgh Pennsylvania USA; ^4^ Department of Environmental and Occupational Health University of Pittsburgh Pittsburgh Pennsylvania USA; ^5^ Center for Vaccine Research University of Pittsburgh Pittsburgh Pennsylvania USA; ^6^ Department of Geology and Planetary Science University of Pittsburgh Pittsburgh Pennsylvania USA; ^7^ Department of Anesthesiology and Perioperative Medicine University of Pittsburgh Pittsburgh Pennsylvania USA; ^8^ Present address: Department of Medicine University of Pittsburgh Pittsburgh Pennsylvania USA; ^9^ Present address: Medicine Institute Allegheny Health Network Pittsburgh Pennsylvania USA; ^10^ Present address: R.D. 2 Portersville Pennsylvania USA; ^11^ Present address: Department of Pathology University of Wisconsin Madison Wisconsin USA

**Keywords:** bacteria, H1N1, influenza, pneumonia, Zinc, Zinc deficiency

## Abstract

Although zinc deficiency (secondary to malnutrition) has long been considered an important contributor to morbidity and mortality of infectious disease (e.g. diarrhea disorders), epidemiologic data (including randomized controlled trials with supplemental zinc) for such a role in lower respiratory tract infection are somewhat ambiguous. In the current study, we provide the first preclinical evidence demonstrating that although diet‐induced acute zinc deficiency (Zn‐D: ~50% decrease) did not worsen infection induced by either influenza A (H1N1) or methicillin‐resistant staph aureus (MRSA), Zn‐D mice were sensitive to the injurious effects of superinfection of H1N1 with MRSA. Although the mechanism underlying the sensitivity of ZnD mice to combined H1N1/MRSA infection is unclear, it was noteworthy that this combination exacerbated lung injury as shown by lung epithelial injury markers (increased BAL protein) and decreased genes related to epithelial integrity in Zn‐D mice (surfactant protein C and secretoglobins family 1A member 1). As bacterial pneumonia accounts for 25%–50% of morbidity and mortality from influenza A infection, zinc deficiency may be an important pathology component of respiratory tract infections.

The authorship order changed due to addition of an author in the revised manuscript.

## 1 INTRODUCTION

Almost half of worldwide deaths in children are attributable to infectious diseases of which pneumonia (18%) and diarrhea (10%) were the leading causes in 2012 (Bhutta & Black, 2013; Salam et al., 2015). Zinc deficiency, estimated to be as high as 20% worldwide, is a common component of overlapping social determinants (poverty and undernutrition) of the aforementioned deaths. Furthermore, zinc has emerged as a single micronutrient, significantly contributing to childhood morbidity and mortality in less developed countries (Penny, 2013; Salam et al., 2015; Wessells & Brown, 2012; Wuehler et al., 2005). Randomized controlled trials with zinc supplementation remain the gold standard for confirming the role of zinc deficiency in epidemiologic studies, especially in developed countries (Hambidge & Krebs, 1999). This has led to Zn supplementation along with oral rehydration as standard medical care for diarrheal disease in children under five (Lamberti et al., 2013). There is considerable ambiguity with respect to zinc supplementation for lower respiratory tract infections (Basnet et al., 2012, 2015; Fu et al., 2013; Howie et al., 2018; Luabeya et al., 2007; Sakulchit & Goldman, 2017; Tie et al., 2016; Wadhwa et al., 2013). Thus, zinc supplementation is not formally a component of prevention (not even as a supplement to available vaccines) or therapy for lower respiratory tract infections.

Preclinical studies are a useful platform to address issues of zinc deficiency and pneumonia in a less ambiguous fashion than the challenges of epidemiologic field studies. Plausibility and underlying mechanisms to support additional human studies can emerge. Previous studies in rodents showed: (a) mice fed a diet low in Zn were increasingly sensitive to *Streptococcus pneumoniae* infection, polymicrobial sepsis (i.e., cecal ligation and puncture), and the parasite *B. microti*; and (b) lungs of alcohol‐induced zinc‐deficient rats were less capable of clearing *Klebsiella pneumoniae* (Hamaguchi et al., 2011; Knoell et al., 2009; Mehta et al., 2011; Strand et al., 2001). Other studies revealed zinc‐deficient rodents had significantly greater acute lung injury secondary to aseptic stimuli including hyperoxia or high tidal volume mechanical ventilation (Boudreault et al., 2017; Taylor et al., 1990). We were particularly interested in the effect of zinc deficiency on influenza A as (a) H1N1 or its related strains, H2N2 and H3N2, or the triple reassortment swine H1N1, were responsible for four well‐documented influenza pandemics in the last 100 years; (b) it is the most common strain in humans, infecting up to 20% of the population and approximately 30,000 associated deaths in US each year; (c) infects respiratory epithelium and replicates rapidly causing endoplasmic reticulum stress and apoptotic death; and (d) altered immune status in zinc deficiency may be critical in viral infections including H1N1 (Buchweitz et al., 2007; Haase & Rink, 2009a; Ludwig et al., 2006; Roberson et al., 2012; Ross et al., 2011; Rynda‐Apple et al., 2015; Sandstead & Prasad, 2010). In addition, the common cause of severe influenza A pathogenesis is superinfection with bacterial pathogens, namely *Staphylococcus aureus* and *S. pneumoniae* (Robinson et al., 2015). Therefore, in the current study, we utilized a mouse model of combined influenza A adapted for mouse (A/PR/8/34) and methicillin‐resistant *S. aureus* (MRSA) infection (McHugh et al., 2013). We note that moderately zinc‐deficient mice (~50% decrease in plasma Zn) were not more sensitive to either influenza A H1N1 or MRSA alone, but were significantly more sensitive than zinc‐replete mice to influenza‐associated bacterial superinfection. As zinc homeostasis may be an important component of host defense with RNA viruses (and their superinfection) in general (Wessels et al., 2020), we discuss our findings in the context of emergent COVID‐19 pandemic and our experience to date with zinc supplementation and infection with SARS‐CoV‐2.

## 2 RESULTS

### 2.1 Zinc deficiency and aerosolized H1N1

**FIGURE 1:**
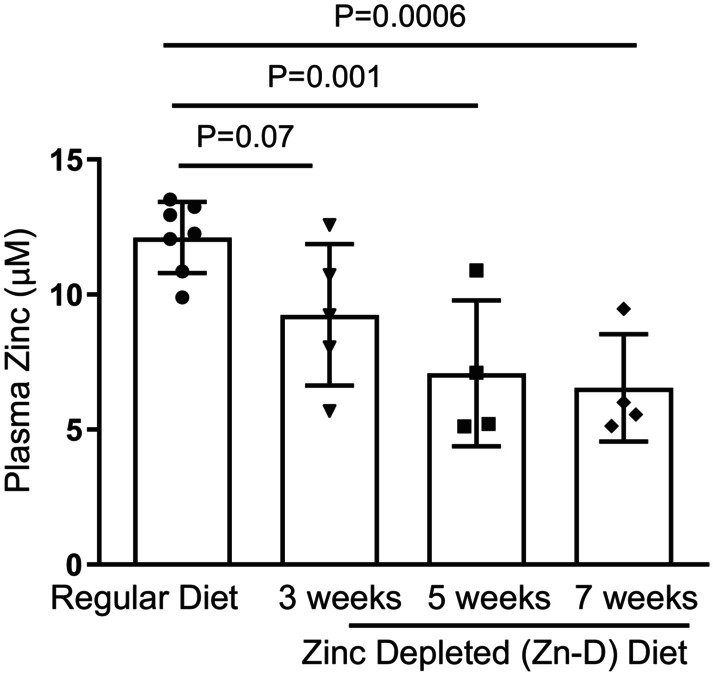
Nutritional induced Zn deficiency. Mice were placed on zinc‐repleted (Zn‐R) or zinc‐depleted (Zn‐D) feed for 7 weeks, and the serum Zn levels were determined on 3, 5, and 7 weeks after the start of the diet by ICP‐MS. Significance was tested by one‐way ANOVA. **p < 0.01, ***p < 0.001, ns‐not significant. Each experiment was independently performed two or more times, and the representative data shown from independent experiments. Values mean ± SD, *n* = 4–7/group.

Mice were placed on a zinc‐deficient diet, and plasma levels of zinc were measured over subsequent 7 weeks (Figure 1). Plasma levels of zinc‐replete (Zn‐R) mice were 12.1 +/− 1.3 μM and decreased by approximately 40% to 7.1 +/− 2.7 μM at 5–7 weeks in zinc‐deplete (Zn‐D) chow. This latter interval of steady‐state zinc deficiency corresponded to the two‐week interval mice studied after exposure to either inhaled H1N1 or H1N1 (oropharyngeal) followed by MRSA.

**FIGURE 2:**
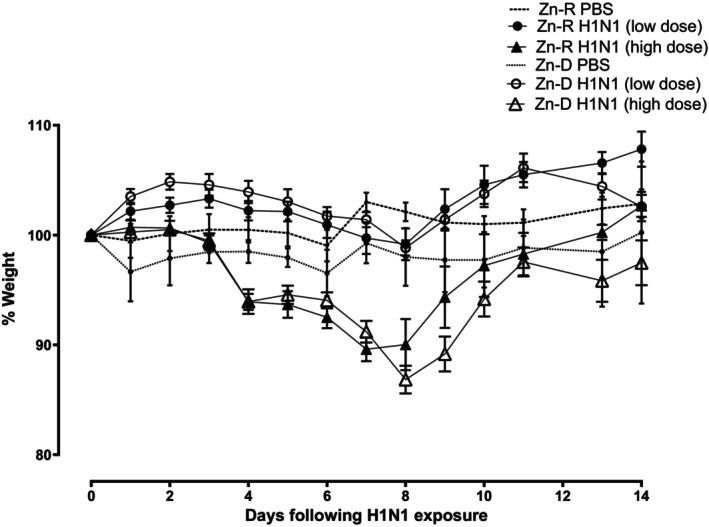
Zinc depletion does not affect change in body weight after H1N1. Mice were placed on Zn‐R or Zn‐D diet for 5 weeks, treated with two different doses of H1N1, and determined the weights from 0 to 14 days following H1N1 treatment. Values are % baseline mean ± SD, *n* = 6–10/group.

Zn‐R and Zn‐D mice were exposed to aerosolized H1N1 (cell‐culture adapted /California/04/2009) in two separate doses (TCID_50_ of 331 or 5478), and changes in body weight were followed for 2 weeks. Within each viral exposure dose group, Zn‐D or Zn‐R status did not affect changes in body weight after exposure to H1N1. The low dose did not affect body weight over the entire two‐week observation period. The higher dose decreased body weight by almost 20% (maximal allowable change) (Figure 2). There were no deaths in this latter group. In separate control groups (i.e., aerosolized PBS only), body weight was similar in either Zn‐R or Zn‐D, and weight was not affected in either group over a two‐week interval (data not shown).

**FIGURE 3:**
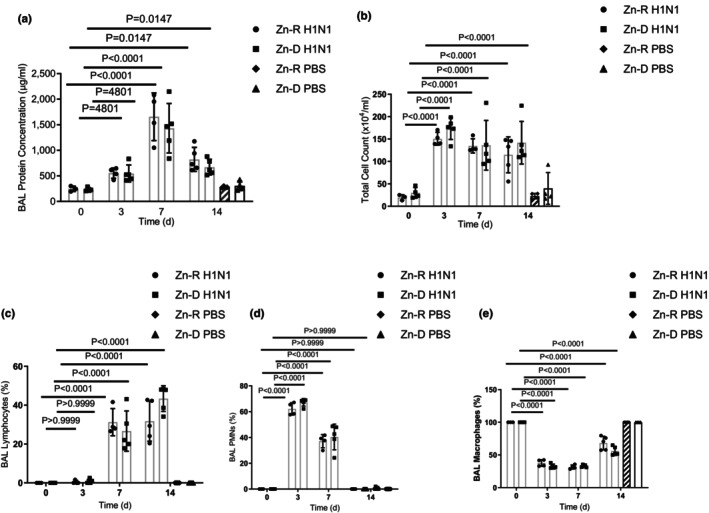
Zinc depletion does not increase the sensitivity to H1N1. Mice were placed on Zn‐R or Zn‐D diet for 5 weeks, treated with H1N1, and determined the BAL protein content, total BAL cell counts, and the differential counts percentage of lymphocytes, polymorphonuclear neutrophils, and macrophages were determined (a–e). Significance was tested by one‐way ANOVA. *p < 0.5, **p < 0.01, ***p < 0.001, ns‐not significant. Each experiment was independently performed two or more times, and the representative data shown from independent experiments. Values mean ± SD, *n* = 4–6/group.

We also determined protein and cell counts/differential cell counts in bronchoalveolar lavage as additional phenotypic descriptions from high‐dose H1N1 infection in Zn‐R and Zn‐D mice (Figure 3). There were no significant differences between Zn‐R and Zn‐D mice in total protein or cellular counts or their differential percentages of neutrophils, macrophages or lymphocytes prior to H1N1 exposure. However, after H1N1 exposure, the following differences were observed: (a) BAL protein increased at day 3 after H1N1 and reached a peak by day 7 and was still significantly greater than pre‐exposure levels at day 14; (b) Total cellular counts increased at day 3 and remained at these high levels in either group for the 2 week interval; (c) each group had a similar neutrophilic infiltration early in the course of influenza followed by a change to larger percentage of lymphocytes; and (d) the flux of these migratory inflammatory/immune cells resulted in an initial decrease in percentage of macrophages that returned towards control by day 14 (Figure 3).

### 2.2 Zinc deficiency does not affect sensitivity to either H1N1 (oropharyngeal) or MRSA, but zinc‐deficient mice are sensitive to combined infection

**FIGURE 4:**
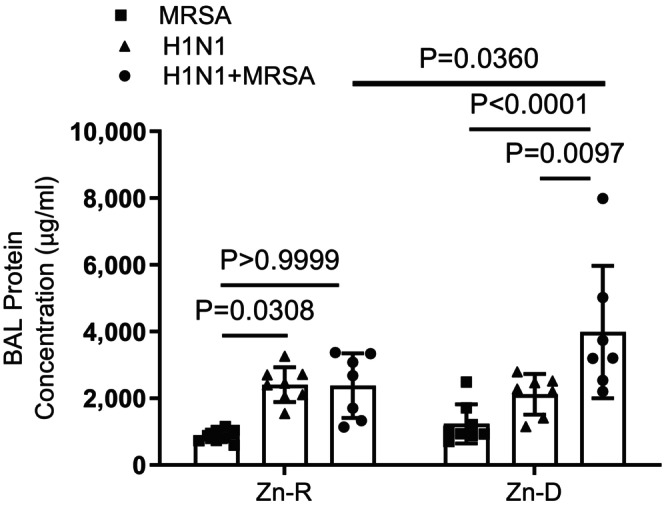
Zinc depletion augments acute lung injury following H1N1, MRSA, and combined infection. Mice were placed on Zn‐R or Zn‐D diet for 5 weeks, treated with H1N1, MRSA, and a combined infection in which the mice were infected with H1N1 for 6 days prior to MRSA infection, and the BAL protein content and total cell counts were measured. Significance was tested by one‐way ANOVA. *p < 0.5, **p < 0.01, ***p< 0.001, ns‐not significant. Each experiment was independently performed two or more times, and the representative data shown from independent experiments. Values mean ± SD, *n* = 7–8/group.

We performed the infection with individual or combined pathogens in Zn‐R mice and then contrasted this to Zn‐D mice. In Figure 4, we show that MRSA or H1N1 alone caused a similar protein leak in Zn‐D mice compared to Zn‐R mice; however, the combination of MRSA and H1N1 significantly increased BAL protein in Zn‐D mice when compared to Zn‐R mice. These data suggest that there is possibly increased lung leakage due to the lung injury in Zn‐D mice.

We further analyzed the BAL differential cell counts and found that there were no significant differences in the percentage of neutrophils, macrophages, and lymphocytes between Zn‐R and Zn‐D mice from individual or combined infections. (Figure S1). We further analyzed the bacterial load in the lung from MRSA and combined infections. The bacterial load was significantly higher in combined‐infected mice compared to MRSA‐infected mice in both Zn‐R and Zn‐D groups. However, no significant differences were observed in bacterial load between Zn‐D and Zn‐R mice in either MRSA or combined infection. (Figure S2). These results suggest that Zn deficiency enhances acute lung injury despite no increase in bacterial load or recruitment of inflammatory/immune cells in combined infections.

Following influenza infection, epithelial cells induce a variety of cytokines and chemokines that attract macrophages and neutrophils to clear the virus (Skoner et al., 1999). Viral infection also triggers the apoptosis of alveolar epithelial cells, which disrupts tight junctions (Atkin‐Smith et al., 2018; Short et al., 2016). Together, these host responses result in lung leakage, bringing more immune cells from the circulation, resulting in a cytokine storm, which is the major cause of pathology due to influenza infection (Kalil & Thomas, 2019).

In this study, we determined whether Zn deficiency impacts the expression of pro‐inflammatory cytokines and chemokines in the lung in the MRSA, H1N1, and combined infection groups. We found no significant differences in the expression of IL‐6, TNFa, CCL2, and CCL5 in the H1N1, MRSA, or combined infection groups between Zn‐D and Zn‐R mice (Figure S3). These data suggest that despite the increase in BAL protein in combined infection, the induction of proinflammatory immune responses in the absence of Zn in H1N1 and MRSA combined infection did not differ.

**FIGURE 5:**
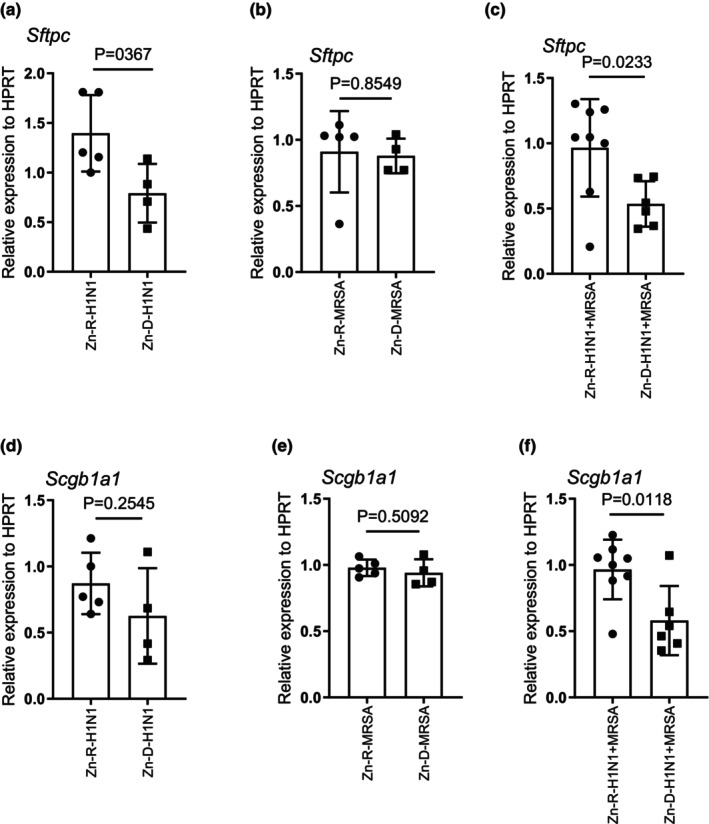
Zinc depletion decreases genes related to epithelial integrity in Zn‐D mice following H1N1, MRSA, combined infection. Mice were placed on Zn‐R or Zn‐D diet for 5 weeks, treated with H1N1, MRSA, and combined infection in which the mice were infected with H1N1 for 6 days prior to MRSA infection, and determined the gene expression of Sftpc and Scgb1a1 by RT‐PCR (a–f). Significance was tested by one‐way ANOVA. *p < 0.5, **p < 0.01, ***p < 0.001, ns‐not significant. Each experiment was independently performed two or more times, and the representative data shown from independent experiments. Values mean ± SD, H1N1 (N = 4–5 per group), or MRSA (*N* = 4–5 per group), H1N1 + MRSA (*N* = 6–8 per group).

Pulmonary surfactants are known to contribute to the host immune response to pulmonary pathogens. Surfactant protein C is expressed in pulmonary type II cells, and studies have shown that SP‐C has a protective role against pulmonary pathogens (Glasser & Mallampalli, 2012). SP‐C can also be used as a surrogate for type II cell integrity (Cipolla et al., 2022; Garcia et al., 2020; Glasser et al., 2013; Pociask et al., 2013). In this study, we determined whether Zn deficiency had any effect on the expression of SP‐C (Sftpc) in response to H1N1, MRSA, and the combined infection. We found a decrease in the expression of Sftpc in the lungs of Zn‐D mice when compared to Zn‐R mice in response to H1N1 and MRSA combined infections; however, we found no differences in the expression of Sftpc in H1N1 or MRSA‐infected mice (Figure 5a–c). These data suggest there is a possible increase in type II cell lung injury due to the absence of Zn in combined infection.

Studies have shown that the lung secretoglobin, secretoglobin family 1A member 1 (Scgb1a1), plays a protective role in the immune response to influenza infection by decreasing the proinflammatory immune responses (Xu et al., 2020). Further, Scgb1a1 is a marker for intact club cell function in the airways. In our study, we found a decrease in the expression of Scgb1a1 in Zn‐D mice when compared to Zn‐R mice in response to H1N1 and MRSA combined infections. Also, we found no differences in the expression of Scgb1a1 in mice infected with H1N1 or MRSA individually (Figure 5d–f). These data suggest that Zn plays a role in preserving expression of Scgb1a1 despite not impacting the expression of proinflammatory cytokine responses. The decrease in Sftpc and Scgb1a1 may be due to increased lung injury due to Zn‐D in the combined infection.

## 3 DISCUSSION

Although zinc deficiency (secondary to malnutrition) has long been considered an important contributor to morbidity and mortality of infectious disease, epidemiologic data (including randomized clinical trials with supplemental zinc) its role in lower respiratory tract infection are somewhat ambiguous. This has important implications for public health and health care delivery in less well‐developed countries. In particular, although zinc supplementation along with rehydration is standard care for diarrheal disorders, routine administration of zinc supplements for pneumonia (bacterial, viral, or fungal) has not been advocated. In the current study, we provide the first preclinical evidence demonstrating that acute zinc deficiency (~40% decrease; Figure 1) does not sensitize mice to H1N1 infection (Figures 2, 3, 4). A low dose of MRSA: (a) in itself caused equivalent modest injury in zinc replete and deplete mice; (b) did not exacerbate H1N1 lung leak in zinc replete mice; but (c) resulted in significantly enhanced lung leak and injury after H1N1 infection in zinc deficient mice (Figure 4). Although the mechanism underlying this latter combined H1N1/MRSA infection is unclear, it was noteworthy that this combination exacerbated injury (Figure 5).

### 3.1 Clinical epidemiology of zinc deficiency and lower respiratory tract infection

The pleiotropic role (structural, catalytic, and signaling) of zinc in innate and adaptive immunity and the likelihood that 1 in 5 persons worldwide may be zinc deficient underscore long‐standing efforts to assess the role of zinc deficiency in infectious disease (Brieger et al., 2013; Haase & Rink, 2009b, 2014a, 2014b; John et al., 2010; Wessells & Brown, 2012; Wessels et al., 2017; Wuehler et al., 2005). Community‐based, double‐blind, controlled trials of zinc supplements are the gold standard in such studies (Hambidge & Krebs, 1999). Since the original study by Black and colleagues, the outcomes of zinc supplementation in significantly reducing morbidity and mortality of diarrhea disorders formed the evidential basis for including zinc supplementation (along with rehydration) in treatment of these disorders (Bhutta et al., 2013; Bhutta & Black, 2013; Salam et al., 2015; Sazawal et al., 1995; Walker et al., 2013). Underscoring these studies is the positive feedback pathophysiologic basis in diarrhea illness including: (a) zinc deficiency, per se, causing diarrhea; and (b) diarrhea leading to excess zinc loss and exacerbation of deficiency (Sazawal et al., 1995).

In contrast, studies that have measured the therapeutic effect of zinc supplementation on outcomes from lower respiratory tract infections are somewhat conflicting (Basnet et al., 2015). A pooled analysis of early trials revealed a large 41% reduction in the incidence of pneumonia with zinc supplementation (Bhutta et al., 1999). A reassessment and meta‐analysis using cause‐specific mortality and morbidity according to the guidelines of Child Health Epidemiology Reference Group for the Lives Saved Tool model indicated a 15% reduction in pneumonia mortality (Yakoob et al., 2011). Nonetheless, a great deal of difference in these studies has precluded incorporating zinc supplementation in prevention and/or therapy of pneumonia (Basnet et al., 2012, 2015; Fu et al., 2013; Howie et al., 2018; Luabeya et al., 2007; Sakulchit & Goldman, 2017; Tie et al., 2016; Wadhwa et al., 2013). Among the limitations of such studies is the use of stunting as a surrogate for zinc deficiency without biomarker status, identification of microbial agent(s), accuracy of morbidity, cause of death endotypes in various registries and/or self‐reporting, unaccounted comorbidities, and various heterogeneities within and between populations. Age‐ and gender‐differences also contribute to measured outcomes.

In addition, most of these studies do not distinguish the cause of pneumonia (viral, bacterial, or fungal), although the relationship between zinc deficiency and altered immune function suggests a role in all forms (Overbeck et al., 2008). We were particularly interested in influenza as its pandemic potential is of significant public health interest, and the potential for zinc deficiency to be a component of sensitivity to H1N1 influenza is apparent (Sandstead & Prasad, 2010). H1N1 was the cause of the first and most recent of four well‐documented influenza pandemics in the last century, and bacterial pneumonia complicated between 25% and 50% of severe infections, with S. pneumonia and *S. aureus* being most complicating organisms (Rynda‐Apple et al., 2015). Accordingly, we were able to utilize our well‐calibrated model of influenza and bacterial superinfection to show that zinc deficiency did not sensitize mice to either H1N1 or MRSA but did show significantly greater lung injury to the combination group (Figure 4) (McHugh et al., 2013; Robinson et al., 2013, 2015).

Our studies provide useful background for the COVID‐19 pandemic and its RNA SARS‐CoV‐2‐induced viral pneumonia. Zn supplementation (with or without the zinc ionophore, hydroxychloroquine; with or without additional nutritional supplements including vitamins C and/or D or selenium) was provided to a sufficient number of patients, which led to case studies (Finzi, 2020), retrospective observational studies (Finzi & Harrington, 2021; Yao et al., 2021), and a description of a randomized case control study (Perera et al., 2020). Meta‐analysis of recent publications suggests some modest benefits of zinc supplementation in hospitalized patients (Abioye et al., 2021) especially if given early during the course of hospitalization, along with vitamin D and selenium (Alexander et al., 2020). A significant concern, as noted in the human studies of Zn supplementation for influenza, is the lack of documenting zinc deficiency in plasma samples of these patients (Khurana et al., 2021). By analogy to influenza, the role of bacterial superinfection in accounting for significant morbidity and mortality in COVID‐19 remains unclear.

### 3.2 Zinc, acute lung injury, immune system, and pneumonia

Surprisingly, in light of the public health importance of placing zinc supplementation in a role as a prevention and/or treatment of lower respiratory tract infections (see above), little is known about the impact of zinc deficiency on pneumonia in controlled animal experiments. Our best insight on zinc deficiency and acute lung injury is an association in sterile lung injuries including hyperoxia and ventilator‐induced lung injury (Boudreault et al., 2017; St Croix et al., 2005; Taylor et al., 1990). Although such injuries involve toll receptor‐mediated pathways and hence a link to innate immunity, a better understanding of zinc deficiency and infection‐mediated lung injury can only be accomplished in infections with live organisms as was shown in zinc‐deficient mice infected (oropharyngeal) with *S. pneumonaiae* (Strand et al., 2001). A study using the paradigm of experimental systemic sepsis (cecal ligation and puncture) revealed greater lung injury (and mortality) in zinc‐deficient mice that was normalized with zinc supplementation (Knoell et al., 2009). Lung injury is often not a component of such polymicrobial sepsis unless the burden of microorganisms is great, suggesting that zinc deficiency indeed sensitized the lung to the histopathologic indices of injury reported by (Knoell et al. (2009) (Iskander et al., 2013). A series of studies in alcohol induced zinc deficiency in rats revealed alveolar epithelial and macrophage dysfunction as well as reduced ability to clear Klebsiella pneumonia (Joshi et al., 2009; Mehta et al., 2011). In a separate study, these authors noted impaired bacterial phagocytosis in alveolar macrophages isolated from human volunteers with alcohol use disorder (Mehta et al., 2013). If administered shortly after the start of the common cold, supplemental zinc (as a homeopathic over‐the‐counter medicine) may reduce the overall course of the disorder in otherwise healthy individuals (Singh & Das, 2013). Pretreatment of mice with zinc reduced mortality and oxygen desaturation in mice (Barnard et al., 2007). We are unaware of any studies in zinc‐deficient mice (or other animals) infected with viruses including influenza (and very currently, SARS‐CoV‐2).

### 3.3 Zinc deficiency and influenza associated‐bacterial pneumonia

In the current study, we noted that diet‐induced zinc deficiency in mice did not sensitize their response to H1N1 influenza. This was true for a low or high dose of H1N1 via aerosol (from no changes in body weight to 20% or maximal permissible loss; Figure 2), and similar neutrophilic response changing to lymphophilic response in the subsequent 2 weeks (Figure 3). In a separate cohort, zinc‐deficient mice responded similarly (Figure 4) to zinc‐replete mice after oropharyngeal administration of H1N1. Although it is possible that a more complete dose response to H1N1 would reveal subtle differences between zinc‐replete and zinc‐deplete animals, it is noteworthy that a large range of doses of H1N1 were investigated via aerosolization, and a separate route of administration (oropharyngeal) of significant amount (enough to increase protein in BAL; Figure 4) was studied. An analogous concern exists for infection with MRSA in that only one modest dose (less toxicity than oropharyngeal H1N1) was studied. The purpose of the current study was not to precisely determine sensitivity of zinc‐deficient mice to bacterial infection alone but rather to focus on combined H1N1‐MRSA infection. In this regard, we carefully calibrated our combined infection model so that the toxicity we observed with H1N1 alone was not exacerbated with MRSA (Figure 4) in zinc‐repleted mice; the combined infection, however, greatly increased acute lung injury (as determined by protein in BAL postmortem and the gene expression of Sftpc and Scgb1a1) in zinc‐depleted mice. Epidemiologic evidence in humans suggests that 25–50% of morbidity or mortality due to H1N1 is due to combined infection. As such, the sensitivity of zinc‐deficient mice to combined H1N1‐MRSA may be informative of human experience in less developed countries. The overall design of these studies was a first attempt to determine the impact of acute diet‐induced zinc deficiency on sensitivity of intact animals to combined viral and bacterial infection and pneumonia. The variety of components of innate and adaptive immunity affected by altered levels of zinc is profound (Wessels et al., 2020), and accordingly, identifying the mechanism(s) underlying such sensitivity is challenging.

We had limitations in our work, first, we will need to expand our injury markers including ATP or LDH in future experiments. Additionally, one logical next step, the zinc repletion will be addressed in future experiment to answer the questions whether zinc repletion after H1N1 or H1N1 + MRSA mitigate the injurious effects of zinc depletion. Such mechanistic experiments would help design better clinical trials where the zinc levels are measured instead of surrogate markers.

In summary, this clinically relevant model of superinfection with H1N1 followed by MRSA suggests the impact of dietary zinc deficiency worsening the clinically relevant H1N1/MRSA infection in mice. Implications for zinc therapy in COVID‐19 await both preclinical assessment and results of randomized controlled trials in humans (Perera et al., 2020).

## 4 MATERIALS AND METHODS

### 4.1 Study overview

Our overall goal was to test whether zinc depletion worsens short‐term survival and augments lung injury in mice exposed to H1N1 alone or in combination with MRSA. Our first goal was to achieve whole body zinc depletion, for which we used zinc‐depleted (Zn‐D) chow (Teklad/Envigo TD.85419, East Millstone, NJ).

For the first part of experiments exploring the relation between zinc and H1N1 infection alone, we purchased (6–8 weeks old), randomly assigned 20 mice to zinc‐depleted (Zn‐D) and 19 mice to zinc‐replete (Zn‐R) diet group. The Zn‐R diet group received a similar caloric content except for the Zn. The mice were fed with a Zn‐D or Zn‐R diet according to the treatment group for 5 weeks. At 5 weeks after diet modification, we exposed mice to inhaled H1N1 and continued to monitor weight and physical activity for additional 2 weeks for a total of 7‐week experimental period. We used an aerosolized H1N1 (2009) model to mimic human Influenza A infection (Toapanta & Ross, 2009). We sacrificed mice after a 2‐week period following influenza exposure by pentobarbital method to assess plasma zinc levels, bronchoalveolar cell distribution, and inflammatory markers.

In the secondary experiments, we fed mice for 5 weeks zinc‐deplete or zinc‐replete chow, then infected 18 mice with H1N1 (PR8) and then 6 days after, infected them with MRSA via the oro‐pharynx (McHugh et al., 2013; Roberson et al., 2012; Robinson et al., 2013). Our previous experience showed a peak of ALI by day 6 or 7 in mice exposed to H1N1 after which the lung injury improves. We therefore exposed mice to secondary bacterial infection by day 6. We sacrificed these mice on day 7 after H1N1 + MRSA exposure. All aspects of this study complied with the Guide for the Care and Use of Laboratory Animals published by the National Institutes of Health (NIH Publication No. 85–23 revised 1996) and met approval of the Institutional Animal Care and Use Committee of the University of Pittsburgh (IACUC 1110706B‐2).

### 4.2 Mice and exposure protocol

C57BL6 wild‐type female mice (age 6–8 weeks) were purchased from the Jackson Laboratory (Bar Harbor, ME). C57BL6 mice were chosen because of their resistance against H1N1 infection (Srivastava et al., 2009). The mice were randomly assigned to either zinc‐replete or ‐deplete diet groups.

### 4.3 H1N1 aerosol inhalation

Aerosols containing Influenza A virus were generated using a Collison 3‐jet nebulizer (BGI, Waltham, MA) controlled by the AeroMP system (Biaera Technologies, Hagerstown, MD) inside a class III biological safety cabinet (Baker Co., Sanford, ME). Aerosol samples were collected in an all‐glass impinger at 6 L/min into 10 mL of DMEM containing 0.5% BSA. Mice were exposed for 10 minutes to aerosolized influenza virus in a whole‐body exposure chamber (Biaera Technologies). Respiratory minute volume was determined for each animal using Guyton's formula, which is based on the weight of the animal [V_m_ = (2.1 × (Weight) × 0.75] (Guyton, 1947). Inhaled dose was calculated by multiplying the respiratory minute volume (V_m_) by the duration of the exposure (*t*) and the aerosol concentration (Ce) (“inhaled dose” = V_m_ × *t* × Ce) (Bowen et al., 2012; Kobasa et al., 2007; Roy et al., 2005).

A high‐titer stock of 2009 H1N1 influenza (A/Ca/04/2009) obtained from BEI Resources (Manassas, VA) was grown using the procedure described in Hancock et al. (2009) and frozen at −80°C until use. Virus titer was determined by Tissue Culture Infective Dose (TCID_50_) assay. Briefly, dilutions of virus were incubated on 96‐well plates containing confluent monolayers of MDCK cells. Cytopathic effect was read at 48 h, and the presence of virus was confirmed by hemagglutination assay with turkey erythrocytes; the TCID_50_ was determined as previously described (Reed, & M., H., 1938).

### 4.4 H1N1 oropharyngeal instillation

Influenza A/PR/8/34 H1N1 was propagated in chicken eggs, as previously described (Braciale, 1977). Mice were infected with 100 PFU Influenza A/PR/8/34 H1N1 (in 40 μL sterile PBS) from a frozen stock or control PBS by oropharyngeal instillation (Gopal et al., 2018; Robinson et al., 2014). The mice were anesthetized and hung by their upper incisors using a sterile thread attached to a solid board. The tongue was gently pulled out using sterile forceps, and the virus (50 μL) was administered to the oropharynx using a micropipette. After 20–30 s of waiting, the mice were gently placed back into the cage. Following administration, the mice were observed for evidence of distress or discomfort and general wellbeing post anesthesia.

### 4.5 MRSA infection

Dr. Alice Prince (Columbia University, New York, NY) (Robinson et al., 2013) provided MRSA (USA 300) (Robinson et al., 2013) as a gift. *S. aureus* was cultured in casein hydrolysate yeast extract–containing modified medium for 18 h to stationary growth phase (Robinson et al., 2013). Mice were inoculated with MRSA (5 × 10^7^ cfu) in 50 μL sterile PBS by oropharyngeal aspiration, and lungs were harvested 24 h later (Lee et al., 2017). Mice in all studies received *S. aureus* 6 days following influenza infection (Robinson et al., 2013). For combined infection, the infected mice were incubated for 6 days and then received *S. aureus* inoculum or control PBS. After an additional 24 h, lungs were harvested (Robinson et al., 2013).The right upper lobe was homogenized in sterile PBS by mechanical grinding for quantification of bacterial burden by plating serial dilutions as described before (Robinson et al., 2013).

### 4.6 Measurement of metal concentrations in mouse plasma

We collected lung tissues into zinc‐free tubes and used titanium instruments to minimize zinc contamination. Whole blood was collected into heparinized tubes, and plasma was separated at 4°C at 10,000 rpm for 5 min. Lung tissues were extracted, and dried lung tissues (∼0.3 g) were digested in concentrated 67–70% nitric acid (Fisher, Fair Lawn, NJ) in Teflon digestion vessels. All of the tubes were prewashed with EDTA and dried in HEPA‐filtered laminar‐flow cabinets configured for trace metal work (AirClean Systems, Raleigh, NC). After centrifugation, the supernatant was diluted 100‐fold with 2% nitric acid (Trace Metal™ Grade Nitric Acid, Thermo Fisher Scientific, Waltham, MA) to ensure a consistent matrix and we measured dilutions by mass. We added internal standard spikes of Be, Ge, and Tl to the diluted solution to minimize the effects of instrument drift during analysis. Metal concentrations (Zn, Cu) in these diluted solutions were measured on a PerkinElmer NexION 300X inductively coupled plasma mass spectrometer (ICP‐MS) (PerkinElmer, Waltham, MA). The instrument was calibrated before each run utilizing a five‐point concentration curve. We assessed drift and measured blanks every ten samples. Diluted solution concentrations were transformed to actual concentrations using the mass measurements recorded during dilution. Detection limits were down to 1 ppt (Lee et al., 2013). Analytical precision was assessed with sample duplicates.

### 4.7 Bronchoalveolar lavage

Total bronchoalveolar lavage (BAL) cell and percentage of differential leukocyte counts were performed at the end of experiments using 1.0 mL phosphate‐buffered saline (PBS). At least 200 cells were counted after the cell smears were made by cytospin and stained with Diff Quick staining (Fisher Healthcare Hema 3 Fixative and solutions, 122,952, 122,937, 122,929). BAL total protein content as a marker of permeability changes was measured by the modified Bradford assay (5,000,002, Bio‐Rad, Hercules, CA) (Robinson et al., 2013).

### 4.8 Rt‐PCR

We extracted RNA from the middle and caudal lung lobes using an RNA isolation kit (400,800, Agilent Technologies, Santa Clara, CA) and converted it to cDNA using a reverse transcription supermix (1,708,841, Bio‐Rad Laboratories, Hercules, CA). Next, we analyzed the gene expression levels of pro‐inflammatory cytokines IL‐6 (Mm00446190_m1), TNFa (Mm00443258_m1), chemokines CCL‐2 (Mm00441242_m1), CCL5 (Mm01302427_m1), lung epithelial surfactant protein C (Sftpc (Mm00488144_m1), and secretoglobins (Scgb1a1 (Mm00442046) by RT‐PCR using TaqMan probes and primers (Thermo Fisher Scientific).

### 4.9 Data analysis

Data are expressed as means ± SD (Lee et al., 2017). A one‐way ANOVA and post‐hoc comparisons were performed. A *p* value of <0.05 was considered significant (Kudva et al., 2011; Lee et al., 2017).

## CONFLICTS OF INTEREST STATEMENT

The authors have no relevant conflict of interest.

### Ethical statements

The study was approved by the Institutional Animal Care and Use Committee of the University of Pittsburgh.

## Supporting information


**Figures S1–S3**.

## Data Availability

The authors confirm that the data supporting the findings of this study are available within the article and its supplementary material.
