# Supplementary material for: Zinc deficiency enhances sensitivity to influenza A associated bacterial pneumonia in mice
Source: Physiol Rep. 2024 Jan 1;12(1):e15902. doi: 10.14814/phy2.15902 (PMC10758336; doi:10.14814/phy2.15902)
Supplement: Supplementary file 1 — Figures S1–S3. [file PHY2-12-e15902-s001.pdf]

Supplementary Figure-1

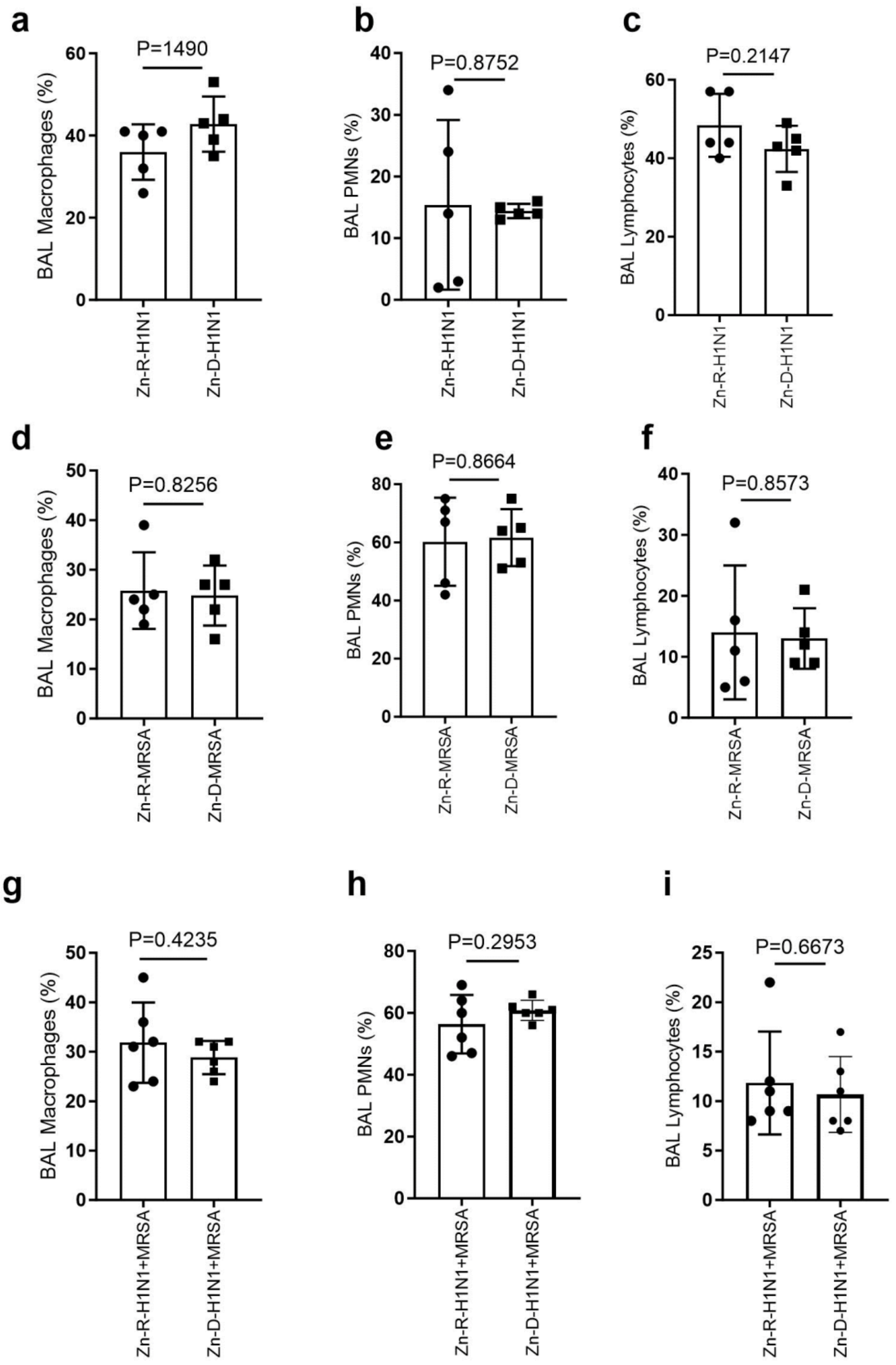

Supplementary Fig.1. Zinc depletion does not affect the percentage of BAL differential cell counts following H1N1, MRSA, and H1N1+MRSA combined infection. Mice were placed on Zn-R or Zn-D diet for 5 weeks, treated with H1N1, MRSA, and combined infection in which the mice were infected with H1N1 for 6 days prior to MRSA infection, and determined the percentage of differential BAL macrophages, neutrophils, and lymphocytes. Significance was tested by one-way ANOVA. *p < 0.5, **p < 0.01, ***p < 0.001, ns-not significant. Each experiment was independently performed two or more times, and the representative data shown from independent experiments. Values mean ± SD, H1N1 (N=5 per group), or MRSA (N=5 per group), H1N1 + MRSA (N=5-6 per group).

Supplementary Figure-2

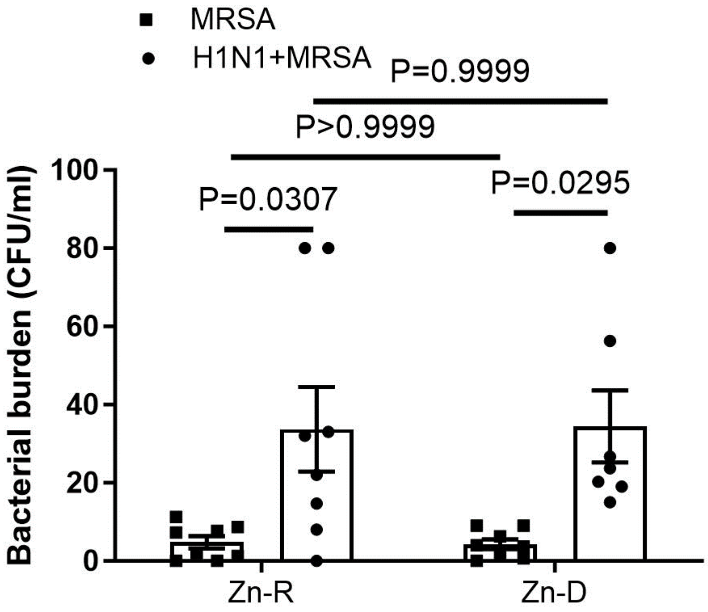

Supplementary Fig.2. Zinc depletion does not affect the bacterial burden following MRSA or H1N1 + MRSA, combined infection. Mice were placed on Zn-R or Zn-D diet for 5 weeks, treated with H1N1, MRSA, and a combined infection in which the mice were infected with H1N1 for 6 days prior to MRSA infection, and the right upper lung lobes were homogenized in PBS, and bacterial burden was determined.

Significance was tested by one-way ANOVA. *$p < 0.5$, **$p < 0.01$, ***$p < 0.001$, ns-not significant. Each experiment was independently performed two or more times, and the representative data shown from independent experiments. Values mean ± SD, MRSA (N=8 per group), H1N1 + MRSA (N=7-8 per group).

Supplementary Figure-3

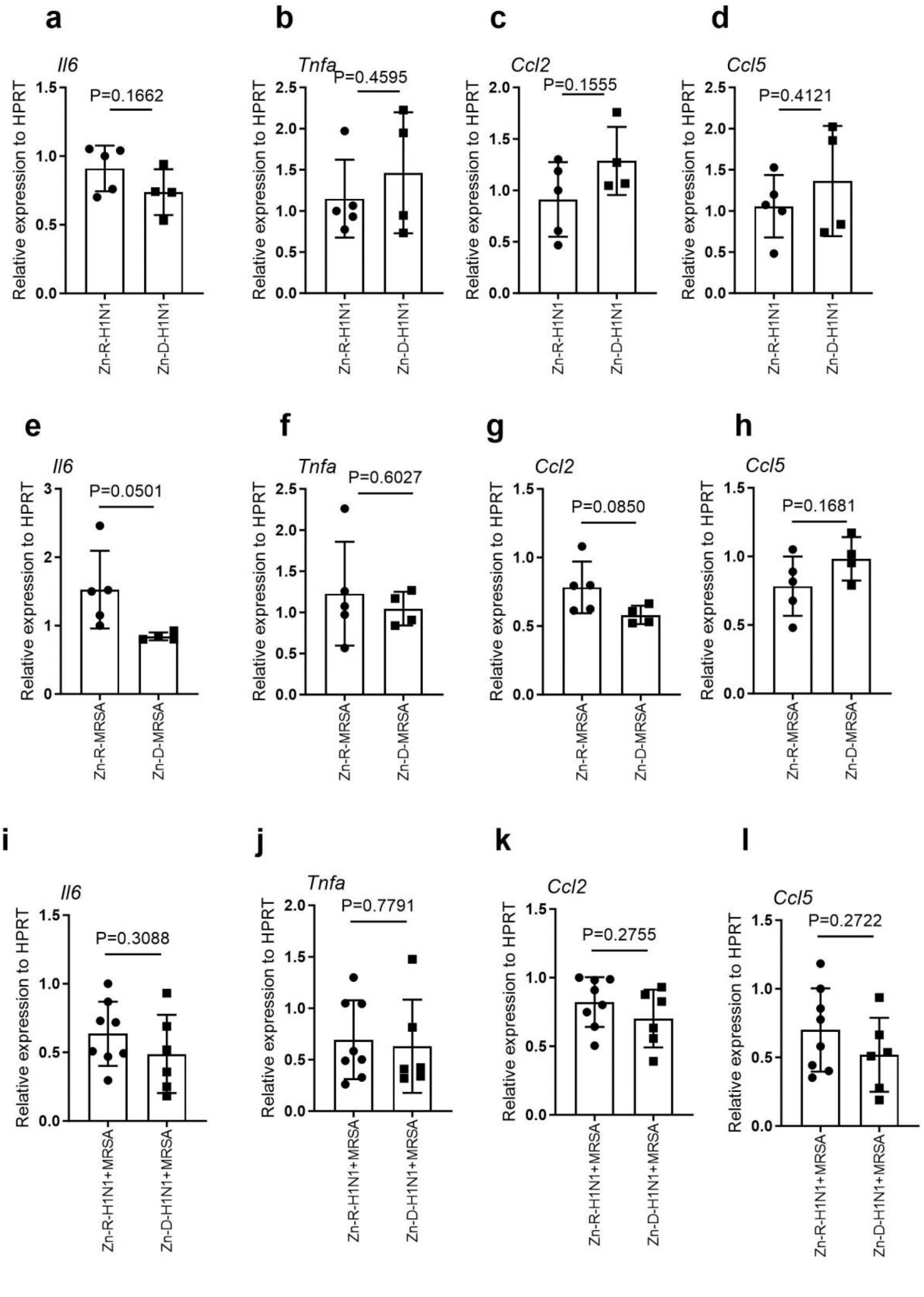

Supplementary Fig.3. Zinc depletion does not affect the expression of proinflammatory cytokines and chemokines following H1N1, MRSA, and H1N1+MRSA combined infection. Mice were placed on Zn-R or Zn-D diet for 5 weeks, treated with H1N1, MRSA, and a combined infection in which the mice were infected with H1N1 for 6 days prior to MRSA infection, and determined the gene expression of IL-6, TNFa, CCL2, and CCL5 by RT-PCR. Significance was tested by one-way ANOVA. *p < 0.5, **p < 0.01, ***p < 0.001, ns-not significant. Each experiment was independently performed two or more times, and the representative data shown from independent experiments. Values mean ± SD, H1N1 (N=4-5 per group), or MRSA (N=4-5 per group), H1N1 + MRSA (N=6-8 per group).
